# A multicenter randomized controlled study of an extracorporeal cytokine hemoadsorption device in septic patients

**DOI:** 10.1186/cc12000

**Published:** 2013-03-19

**Authors:** D Schädler, C Porzelius, A Jörres, G Marx, A Meier-Hellmann, C Putensen, M Quintel, C Spies, C Engel, N Weiler, M Kuhlmann

**Affiliations:** 1 University Medical Center Schleswig-Holstein, Campus Kiel, Germany; 2 University of Leipzig, Germany; 3 Charité University Hospital Campus Virchow-Klinikum, Berlin, Germany; 4 RWTH University Hospital Aachen, Germany; 5 HELIOS Klinikum, Erfurt, Germany; 6 University of Bonn, Germany; 7 University Hospital Göttingen, Germany; 8 Campus Charité Mitte and Campus Charité Virchow-Klinikum, Charité-University Medicine Berlin, Germany; 9 Vivantes Klinikum im Friedrichshain, Berlin, Germany

## Introduction

A novel sorbent hemoadsorption device for cytokine removal (CytoSorbents, USA) was developed and successfully tested in animal models of sepsis. The experience in the clinical setting is still limited to case reports. In this first clinical trial, we tested the hypothesis that treatment with sorbent hemoadsorption could safely and effectively reduce cytokines in septic patients with acute lung injury (ALI).

## Methods

Ventilated patients fulfilling the criteria for severe sepsis and ALI were enrolled in this multicenter randomized, controlled, open-label study comparing standard of care with or without hemoperfusion treatment. Primary endpoints were safety and IL-6 reduction. Treated patients underwent hemoperfusion at flow rates of ~200 to 3003ml/ minute for 6 hours per day for 7 consecutive days. The overall mean reduction in individual plasma cytokines for the control and treatment groups during the treatment period was calculated using a generalized linear model.

## Results

Forty-three patients (18 treated, 25 control) completed the study and were further analyzed. Incidence of organ dysfunction at enrollment (treatment vs. control) was: septic shock (94% vs. 100%, *P *= 0.42), acute respiratory distress syndrome (67% vs. 56%, *P *= 0.33), and renal failure (39% vs. 24%, *P *= 0.54). During 115 treatments no serious device-related adverse events occurred. On average, there were no changes in hematology and other blood parameters except for a modest reduction in platelet count (<10%) and albumin (<5%) with treatment. Hemoperfusion decreased IL-6 blood concentration significantly (-49.1%, *P *= 0.01), with similar reductions of MCP-1 (-49.5%, *P *= 0.002), IL-1ra (-36.5%, *P *= 0.001), and IL-8 (-30.2%, *P *= 0.002). The 28-day mortality (28% vs. 24% control, *P *= 0.84) and 60-day mortality (39% vs. 32% control, *P *= 0.75) did not differ significantly between the two studied groups.

## Conclusion

In this first clinical study of a novel sorbent hemoadsorption device in patients with severe sepsis and ALI, the device appeared to be safe and decreased the blood concentration of several cytokines. Further research is needed to study the effect of the device on the clinical outcome of septic patients.

